# Revealing Potential Spinal Cord Injury Biomarkers and Immune Cell Infiltration Characteristics in Mice

**DOI:** 10.3389/fgene.2022.883810

**Published:** 2022-05-30

**Authors:** Liang Cao, Qing Li

**Affiliations:** ^1^ Department of Traumatic Orthopedics, The Second Affiliated Hospital, University of South China, Hengyang, China; ^2^ School of Clinical Medicine, Guizhou Medical University, Guiyang, China; ^3^ Department of Orthopedics Traumatic, The Affiliated Hospital of Guizhou Medical University, Guiyang, China

**Keywords:** spinal cord injury, immune cells, key genes, bioinformatics analysis, machine learning strategies

## Abstract

Spinal cord injury (SCI) is a disabling condition with significant morbidity and mortality. Currently, no effective SCI treatment exists. This study aimed to identify potential biomarkers and characterize the properties of immune cell infiltration during this pathological event. To eliminate batch effects, we concurrently analyzed two mouse SCI datasets (GSE5296, GSE47681) from the GEO database. First, we identified differentially expressed genes (DEGs) using linear models for microarray data (LIMMA) and performed functional enrichment studies on those DEGs. Next, we employed bioinformatics and machine-learning methods to identify and define the characteristic genes of SCI. Finally, we validated them using immunofluorescence and qRT-PCR. Additionally, this study assessed the inflammatory status of SCI by identifying cell types using CIBERSORT. Furthermore, we investigated the link between key markers and infiltrating immune cells. In total, we identified 561 robust DEGs. We identified Rab20 and Klf6 as SCI-specific biomarkers and demonstrated their significance using qRT-PCR in the mouse model. According to the examination of immune cell infiltration, M0, M1, and M2 macrophages, along with naive CD8, dendritic cell-activated, and CD4 Follicular T cells may have a role in the progression of SCI. Therefore, Rab20 and Klf6 could be accessible targets for diagnosing and treating SCI. Moreover, as previously stated, immune cell infiltration may significantly impact the development and progression of SCI.

## Introduction

Spinal cord injury (SCI) is a devastating injury that frequently results in total or partial impairment of motor, sensory, and sphincter function (Lago et al., 2018). Moreover, whether classified as traumatic or non-traumatic, SCI always causes significant lifelong disability. SCI is becoming more common as vehicle accidents and extreme sports increase. As a result, this condition has disastrous impacts on patients, families, and society (Ropper and Ropper, 2017).

The pathological process of SCI is generally divided into two stages (Alizadeh et al., 2019). The primary injury causes hemorrhage, ischemia, edema, anoxia, and neuron and glial cell necrosis. The secondary injury involves complex pathophysiologic mechanisms, including ionic imbalance, free radical stress, inflammatory responses, and glial scars. Although the creation of glial scars can slow secondary damage spread, it also inhibits axon regrowth. Secondary injuries impair nerve plasticity and functional recovery. The main challenge in SCI treatment development is the difficulty of repairing injured neurons and restoring the conducting function of axons. Currently, no effective drugs or therapeutic approaches exist for SCI (Badhiwala et al., 2019). As many patients experience severe physical and psychological consequences, SCI has become a global issue. Thus, elucidating the specific molecular mechanisms underlying the pathophysiology of SCI is crucial.

Recently, an increasing number of articles revealed that immune cell infiltration plays a pivotal role in SCI healing. For example, microRNA-151-3p is abundant in microglia-derived exosomes and has neuroprotective properties during SCI healing (Li et al., 2021). The chemokine (C-C motif) ligand 28 (CCL28) acts as a protective factor after SCI by recruiting C-C chemokine receptor 10 (CCR10)-positive and immunosuppressive regulatory T cells (Wang et al., 2019). After SCI, interleukin 19 (IL-19) enhances locomotor function recovery and decreases motor neuron loss, as well as microglial and glial activation (Guo et al., 2018). C3 is a novel Th2 interleukin reducing neurite outgrowth and neuronal survival *in vitro* and axon regeneration *in vivo* (Peterson et al., 2017). Chronic SCI can impair CD8 T cell function by up-regulating programmed cell death-1 expression (Zha et al., 2014). γδ T cells are recruited to the SCI site, promoting the inflammatory response and exacerbating neurological impairment. CCL2/CCR2 signaling is critical for T cell recruitment to the SCI site and may be used as a novel therapeutic target in the future (Xu P. et al., 2021). Nonetheless, it is necessary to elucidate the molecular mechanism by which diverse immune cells influence SCI progression. As previously stated, assessing immune cell infiltration and dissecting the components of invading immune cells is crucial for unraveling the SCI molecular system and identifying novel immunotherapeutic targets (Ahmed et al., 2018; Al Mamun et al., 2021). CIBERSORT is a computational method for quantifying cell composition using gene expression data. This approach may help characterize immune cell infiltration (Newman et al., 2015).

We used the GEO database to obtain microarray datasets and conduct differential expression gene analyses. Additionally, we combined bioinformatics analysis and machine-learning techniques to thoroughly screen and identify key SCI genes. Next, we used CIBERSORT to compare immune cell infiltration in 25 immune cell subsets between SCI and sham samples. Additionally, we explored the relationships between the key genes and immune cells to better understand the molecular immunological mechanisms during SCI development.

## Materials and Methods

### Data Source

We downloaded two SCI datasets (GSE5296, GSE47681) from the NCBI Gene Expression Omnibus (GEO) (Clough and Barrett, 2016). These two datasets were gene expression arrays generated using GPL1261 [Mouse430_2] Affymetrix Mouse Genome 430 2.0 Array (Affymetrix, Santa Clara, CA, United States) (Barrett et al., 2013). We selected 18 samples with SCI and 12 sham samples from the GSE5296 dataset. Similarly, we selected 17 spinal cord tissue samples from the GSE47681 dataset (Wu et al., 2013), including 13 samples with SCI and 4 sham spinal cord tissue samples.

### Data Normalization and Differentially Expressed Genes Screening

We processed the two SCI datasets using the R package “affy,” notably for normalization and log2 transformation (Irizarry et al., 2003). Here, we considered the average value as the expression value when a group of probes corresponded to the same gene. Moreover, we eliminated the batch effects between two datasets using the surrogate variable analysis (SVA) package from Bioconductor (Leek et al., 2012). Finally, we screened the DEGs using the LIMMA package with a *p*-value < 0.05 and |log2 Fold change (FC)| > 1 (Ritchie et al., 2015).

### GO, Kyoto Encyclopedia of Genes and Genomes, and GSEA Analysis of the Differentially Expressed Genes

We performed the analysis of Gene Ontology (GO) and Kyoto Encyclopedia of Genes and Genomes (KEGG) for the DEGs using DAVID 6.8 (https://david-d.ncifcrf.gov/). To understand the function of the DEGs, we uploaded them to the DAVID (Ritchie et al., 2015) and KOBAS databases (http://kobas.cbi.pku.edu.cn/) (Wu et al., 2006). We used a *p*-value < 0.05 and count ≥ 2 as the significant enrichment threshold. To provide a more intuitive understanding of the gene expression levels associated with significantly enriched functional pathways, we performed a gene set enrichment analysis (GSEA) using the R software (Subramanian et al., 2005).

### Screening and Validation of Characteristic Genes

We screened for key genes associated with SCI using three algorithms: least absolute shrinkage and selection operator (LASSO) regression analysis (Tibshirani, 1996), random forests analysis (Strobl et al., 2007; Wang et al., 2016), and support vector machine-recursive feature elimination (SVM-RFE) analysis (Suykens and Vandewalle, 1999). For the random forest method, we used the R package “randomForest”. We performed the LASSO logistic regression using the R package “glmnet,” and a lambda of zero was considered optimal. We constructed the SVM classifier with tenfold cross-validation using the R package “e1071.” We also used the RFE function within the “caret” package to select the featured gene using tenfold cross-validation. Then, we selected the genes from the three classification models for further analysis. The GSE45006 dataset was used as a validation dataset (Niu et al., 2021).

### Spinal Cord Injury Procedure and Immunofluorescence

6–8 weeks C57BL/6 mice were obtained from the Experimental Animal Center of Guizhou Medical University [license no. SCXK (Qian) 2018-0001]. All animal experiments were approved by the Animal Care and Use Committee of Guizhou Medical University. We divided the mice into an SCI group and a sham group. All mice were anesthetized with 1.25% Avertin. First, we performed a 1-cm dorsal incision and performed a laminectomy of the T10 vertebra. We crushed the spinal cord with vessel clamping for 15 s. Paralysis of both lower limbs indicated successful modeling. For the sham group, we isolated the skin and tissue to expose the spinal cord without injuring the animals. After surgery, we returned the mice to their home cages and performed manual bladder expression three times a day. We sacrificed the animals 7 days later. We injected 100 ml of phosphate-buffered saline (PBS) from the apex with a syringe to remove the blood, followed by 100 ml of 4% paraformaldehyde for tissue fixation until the mouse body was rigid. Next, we fixed the spinal cord with formalin and embedded it in paraffin before transversely cutting 20-µm-thick tissue sections using a Cryotome. We washed the sections three times with PBS for 5 min and added blocking buffer (10% goat serum and 0.3% TritonX-100) for 1 h. We then incubated the sections with the primary antibody overnight at 4°C, washed them three times with PBS for 5 min, incubated them with secondary antibodies (goat anti-rabbit Alexa Fluor 488,1:500,CST) in the dark for 2 h, and stained cells nuclei with DAPI (4',6-diamidino-2-phenylindole). Finally, we photographed the sections with a laser confocal microscope.

We used Rab20 (YT3922, 1:200, Immunoway) and Klf6 (14716-1-AP, 1:200, Proteintech) as primary antibodies.

### Quantitative PCR Analysis

To summarize, we extracted total RNA from the spinal cord of mice using the TRIZOL reagent (TIANGEN BIOTECH Corp, Beijing, China), then polyadenylated and reverse-transcribed it into cDNA using a poly(T) adapter following the manufacturer’s instructions. We performed real-time PCR using a thermal cycler with the following parameters: a 5 min initial denaturation step at 95°C; 44 cycles at 95°C for 15 s; 55°C for 30 s, and 72°C for 20 s. We subjected each sample to the entire experimental procedure in triplicate. Table 1 lists the primers specific for mRNA.

**TABLE 1:** mRNA-specific primers of key genes.

Gene	Primer	Sequence (5′-3′)	PCR products
Klf6	Forward	GTT​TCT​GCT​CGG​ACT​CCT​GAT	108bp
	Reverse	TTC​CTG​GAA​GAT​GCT​ACA​CAT​TG	
Rab20	Forward	GGG​AGC​AGT​TTC​ATG​GTC​TGG	143bp
	Reverse	GCA​GTC​ATT​GTT​GGC​TGT​TTC	
β-tubulin	Forward	CTG​TCC​GTC​CAT​CAG​TTG​GT	122bp
	Reverse	TGG​TTC​AGG​TCT​CCG​TAG​GT	

### Immune Cell Infiltration Analysis

We measured the relative proportions of immune cells in SCI mouse tissue using the CIBERSORT method to annotate merged expression data and calculate immune cell infiltrations based on mouse tissue expression profiles (Chen et al., 2017). Next, we compared the relative levels of 25 immune cells between the SCI and sham groups. A correlation heatmap, produced using the “corrplot” package, revealed the relationships between 25 types of infiltrating immune cells. Finally, we analyzed and visualized the Spearman correlation between key biomarkers and immune infiltrating cells using the “ggstatplot” and “ggplot2” packages.

## Results

### Identification of Differentially Expressed Genes

**FIGURE 1:**
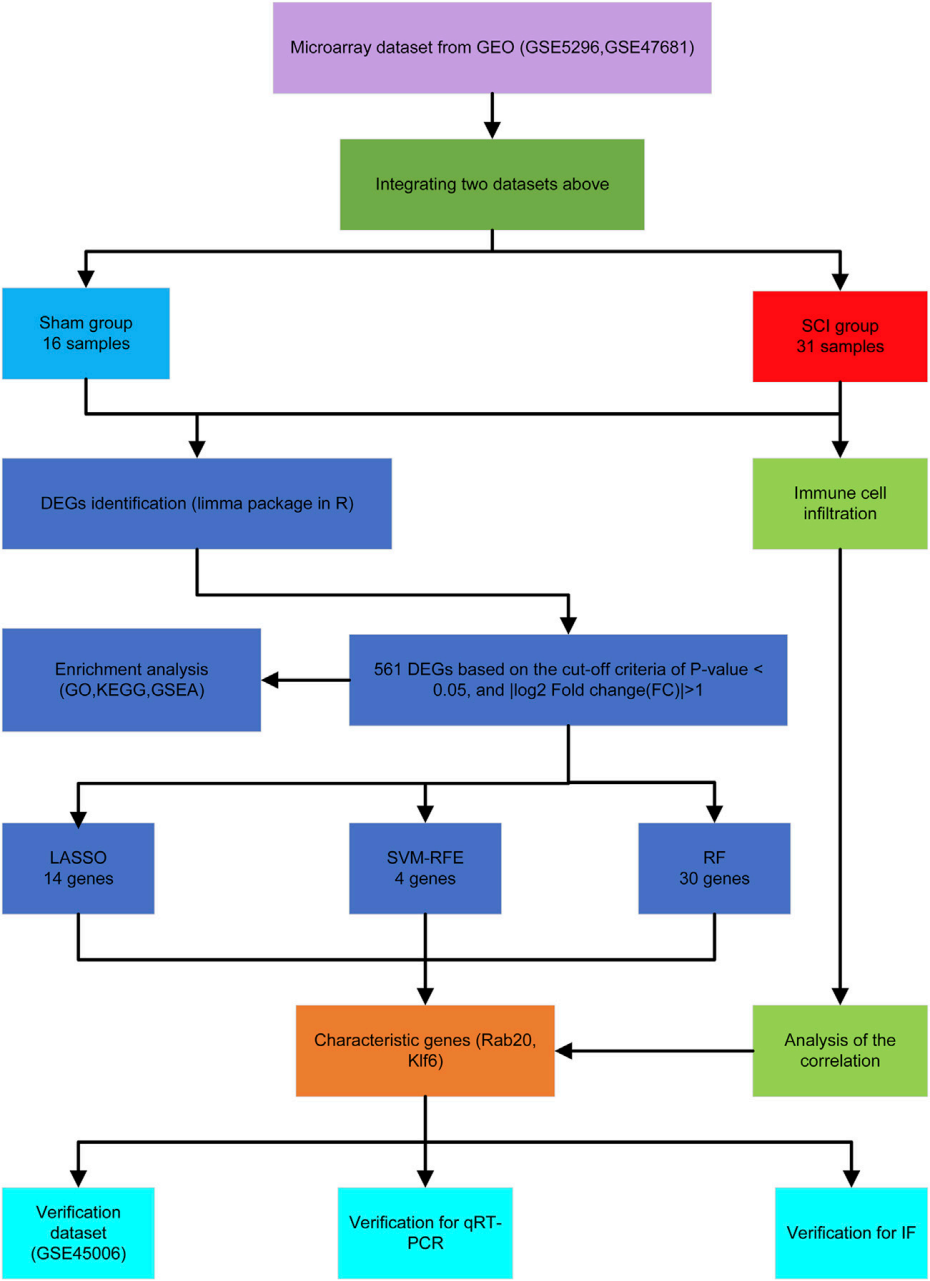
The flowchart of the analysis process.

**FIGURE 2:**
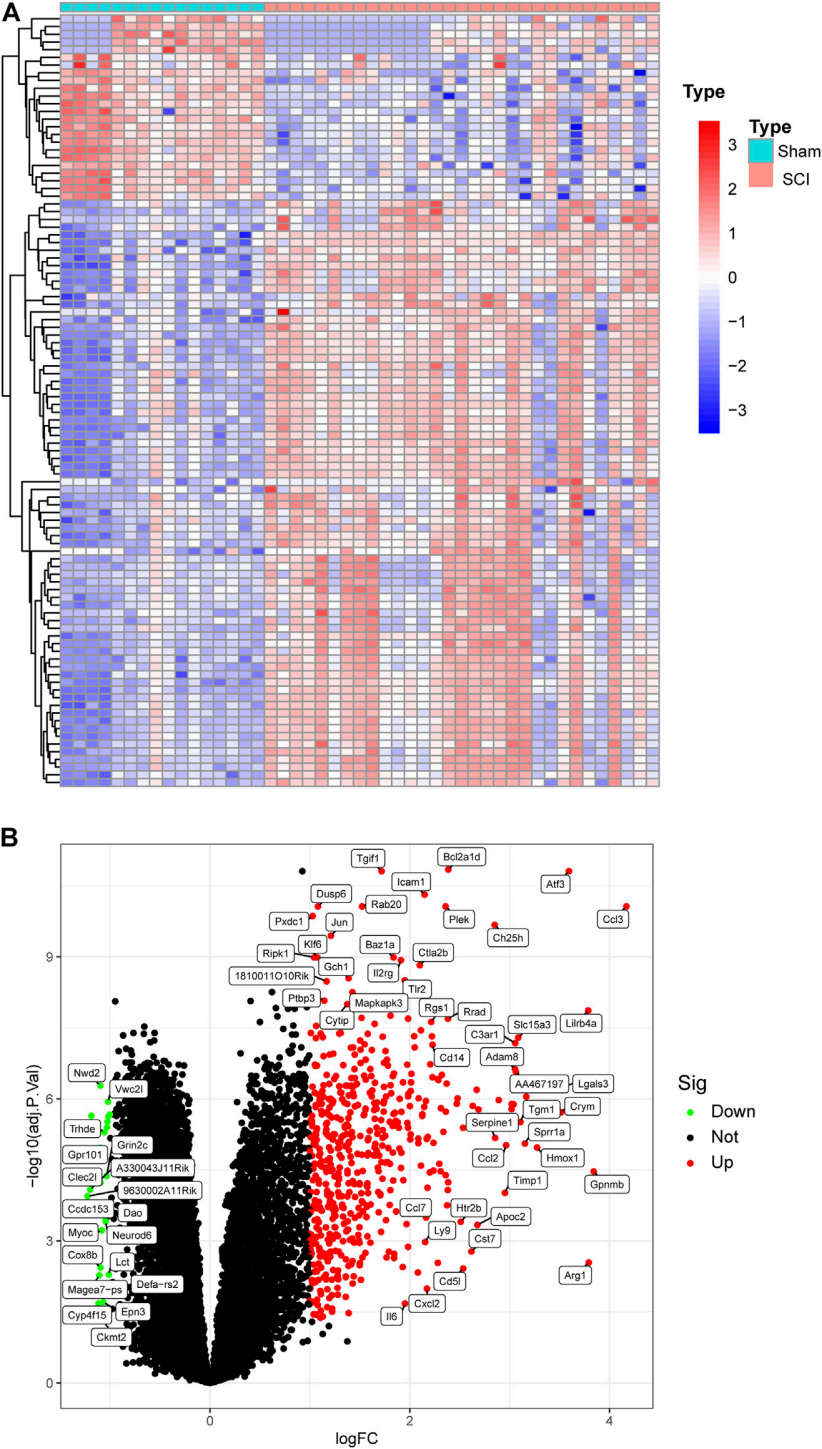
Heat map and Volcano plot of the DEGs. **(A)** Each row of the heat map represented one DEG, and each column represents one sample. The red and blue colors represent upregulated and downregulated DEGs, respectively. **(B)** Red points represented upregulated DEGs, and green points displayed downregulated DEGs.

Figure 1 shows the workflow of this study. We integrated two SCI datasets (GSE5296, GSE47681), including 16 sham samples and 31 SCI samples (Figure 2A). We found a total of 561 DEGs—536 up-regulated genes and 25 downregulated genes. Figures 1B, 2B display the DEGs heatmap and volcano plot, respectively.

### Function Enrichment Analysis

**FIGURE 3:**
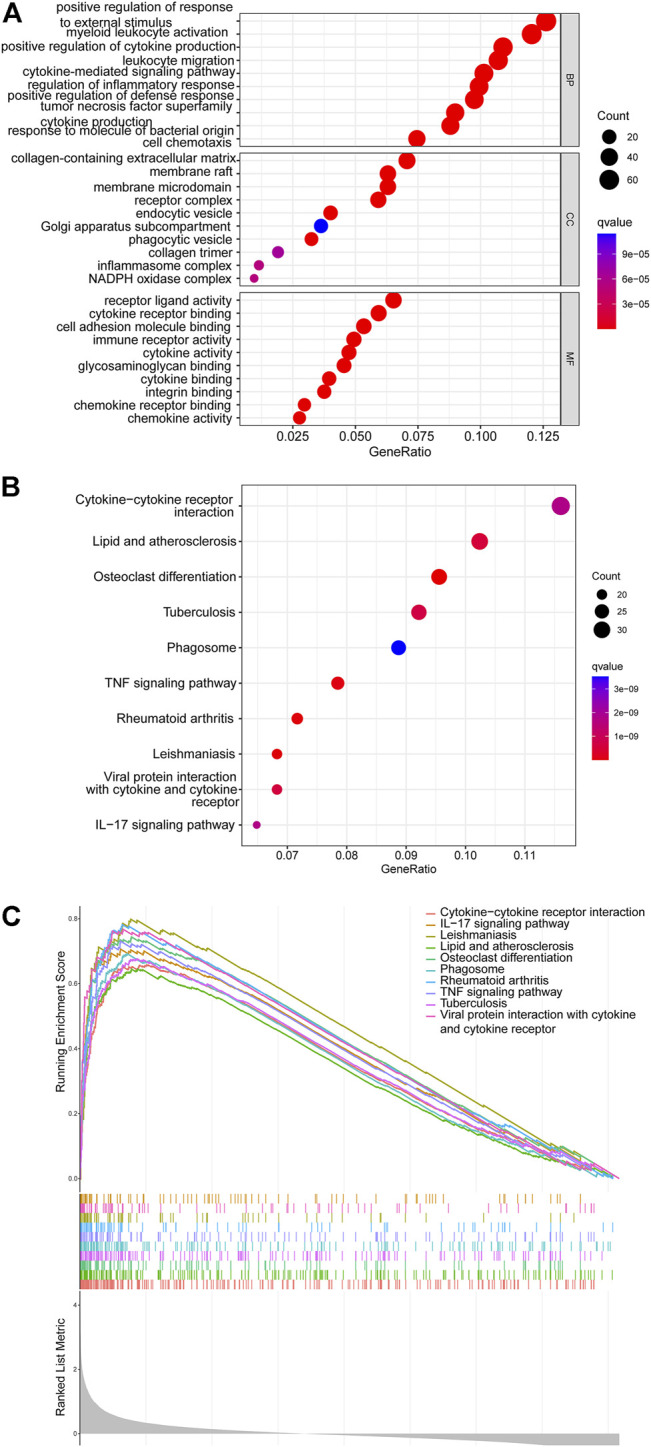
The results of functional enrichment analyses. **(A)** GO analysis results of DEGs. **(B)** KEGG analysis results of DEGs. **(C)** GSEA profiles showed the ten significant GSEA sets.

The GO and KEGG analyses revealed that the DEGs were mainly involved in the biological processes of leukocyte migration, cytokine-mediated signaling pathway, positive regulation of cytokine production, positive regulation of defense response, tumor necrosis factor superfamily cytokine production, response to molecule of bacterial origin, regulation of inflammatory response, and cell chemotaxis. Regarding the cellular components, these DEGs were mainly associated with the membrane raft receptor complex, endocytic vesicle, Golgi apparatus sub-compartment, membrane microdomain, phagocytic vesicle, collagen trimer, collagen-containing extracellular matrix, inflammasome complex, and NADPH oxidase complex (Figure 3A). The KEGG pathway analysis showed that the DEGs were involved in lipid metabolism, cytokine–cytokine receptor interaction, atherosclerosis, osteoclast differentiation, tuberculosis, phagosome, TNF signaling pathway, rheumatoid arthritis, Leishmaniasis, viral protein interaction with cytokine and cytokine receptor, and IL-17 signaling pathway (Figure 3B). Additionally, GSEA data indicated that certain pathways were enriched (Figure 3C). These results suggested that the immune system plays a vital role in SCI.

### Key Biomarkers Screening and Validation

**FIGURE 4:**
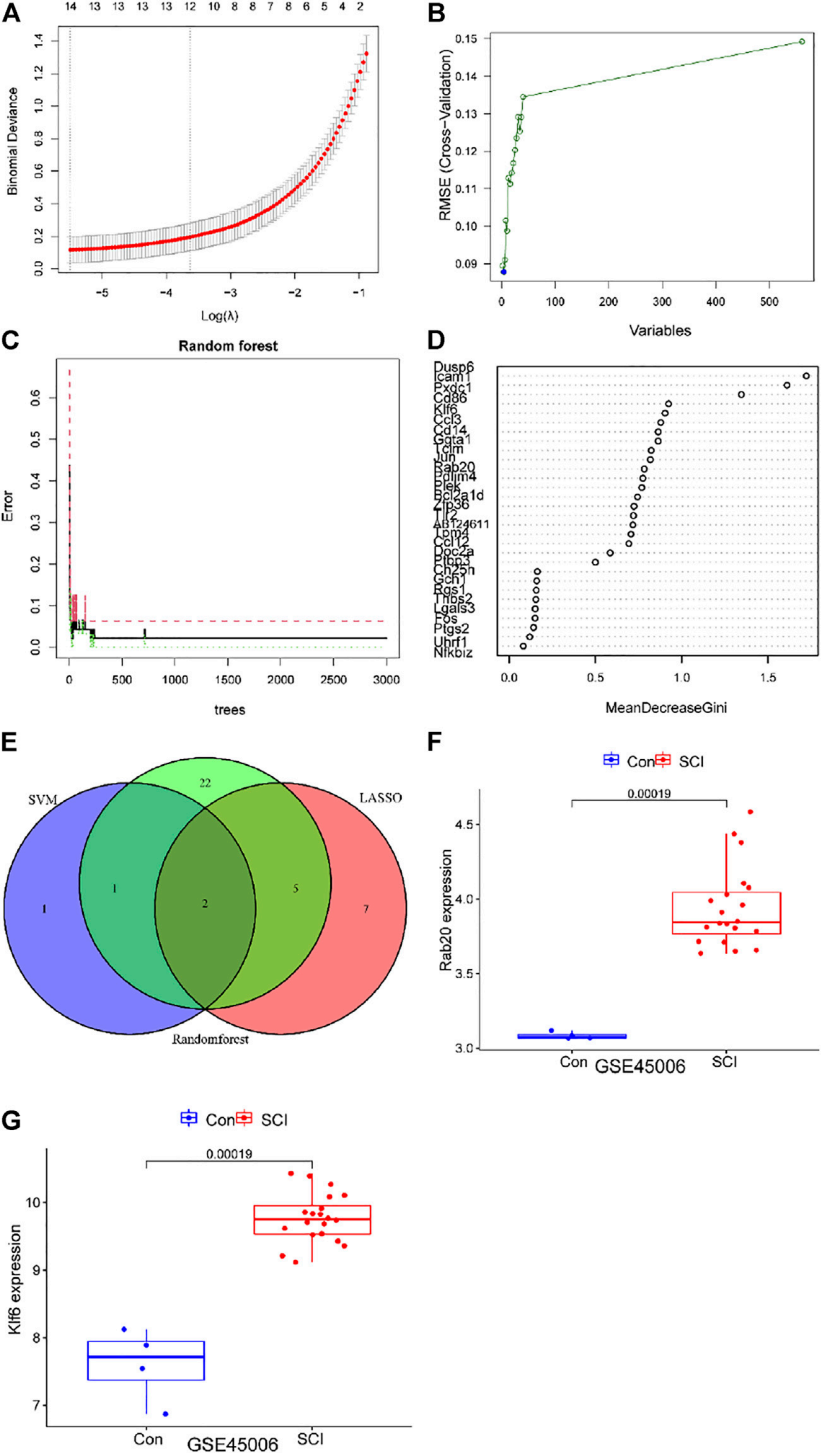
Screening of key genes via the comprehensive strategy. **(A)** screening key markers using Least absolute shrinkage and selection operator (LASSO) logistic regression method. **(B)** screening key markers through support vector machine recursive feature elimination (SVMRFE) method. **(C,D)** random forest (RF) strategy to screen biomarkers. **(E)**Venn diagram displayed the intersection of key markers obtained by the three methods. **(F,G)** The expression levels of Rab20 and Klf6 in GSE45006.

**FIGURE 5:**
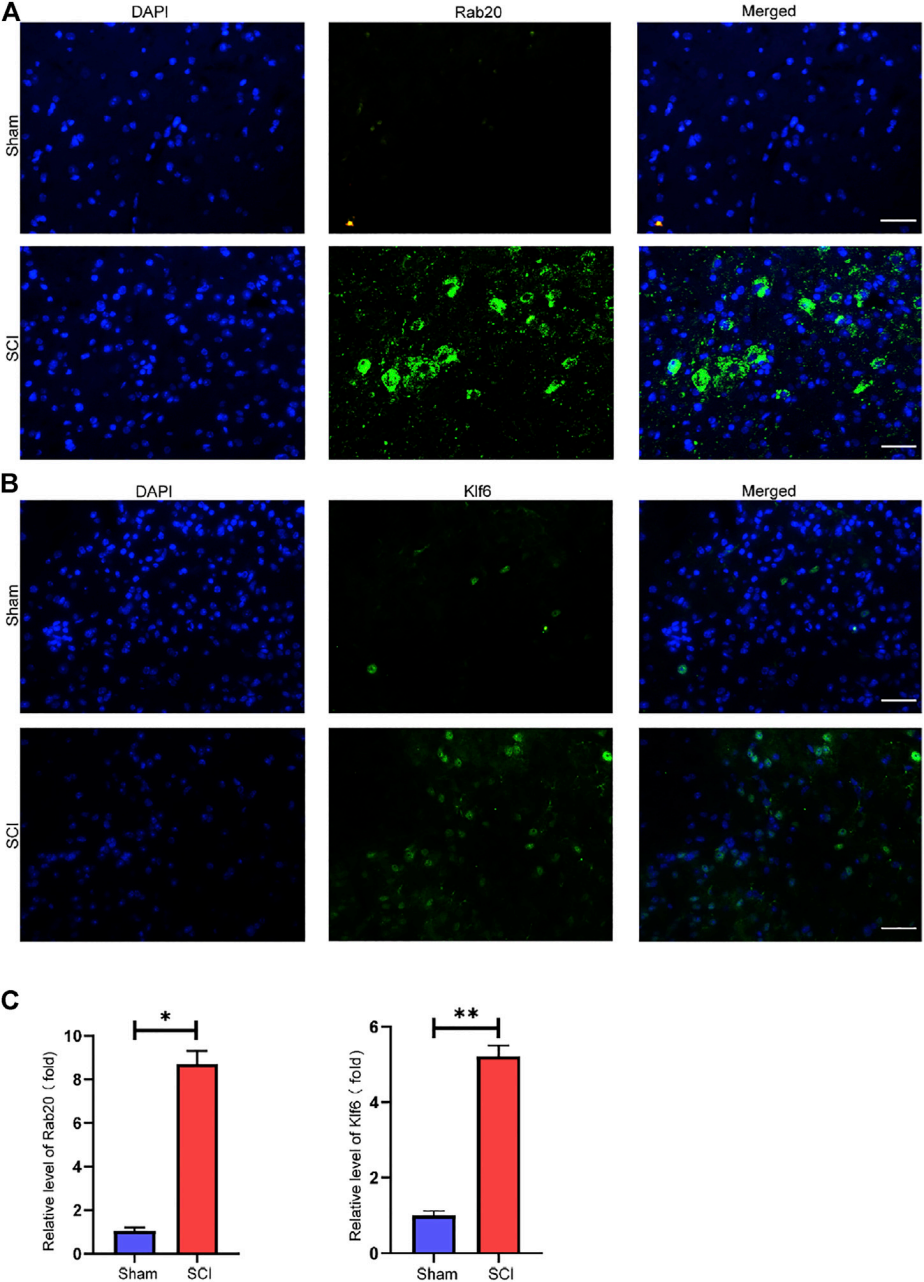
Key genes validation. **(A)** Representative images showed that Rab20 expression was examined using immunofluorescence. **(B)** Representative images showed that Klf6 expression was examined using immunofluorescence. **(C)** qRT-PCR verification of Rab20 and Klf6 in SCI samples of mice and sham samples of mice. The results were represented as mean average ± SE with *p* < 0.05. scale bar represents 100 µM in **(A,B)**.

We used the LASSO logistic regression method to find 14 important biomarkers from the DEGs (Figure 4A). With the SVM-RFE method, we identified four genes qualifying as key biomarkers among the DEGs (Figure 4B). Additionally, we identified 30 genes as significant biomarkers using the random forest strategy (Figures 4C,D). The Rab20 and Klf6 genes were overlapped genes. Thus, we selected Rab20 and Klf6 as key biomarkers for further validation (Figure 4E). To verify the relationship between key genes and SCI vulnerability, we selected GSE45006 as the training data and Rab20 and Klf6 as the test genes. We compared the expression of hub genes during the SCI process. The SCI group had higher Rab20 and Klf6 expression levels than the sham group (Figures 4F,G). To confirm this, we performed immunofluorescence staining experiments using mouse spinal cord tissue. SCI tissues had significantly higher Rab20 and Klf6 levels than those from the sham group (Figures 5A,B). Finally, we quantified Rab20 and Klf6 expression in mouse samples using qRT-PCR. The SCI group had considerably higher levels of these two biomarkers (Rab20 and Klf6) than the sham group (Figure 5C).

### Immune Cell Infiltration Analysis

**FIGURE 6:**
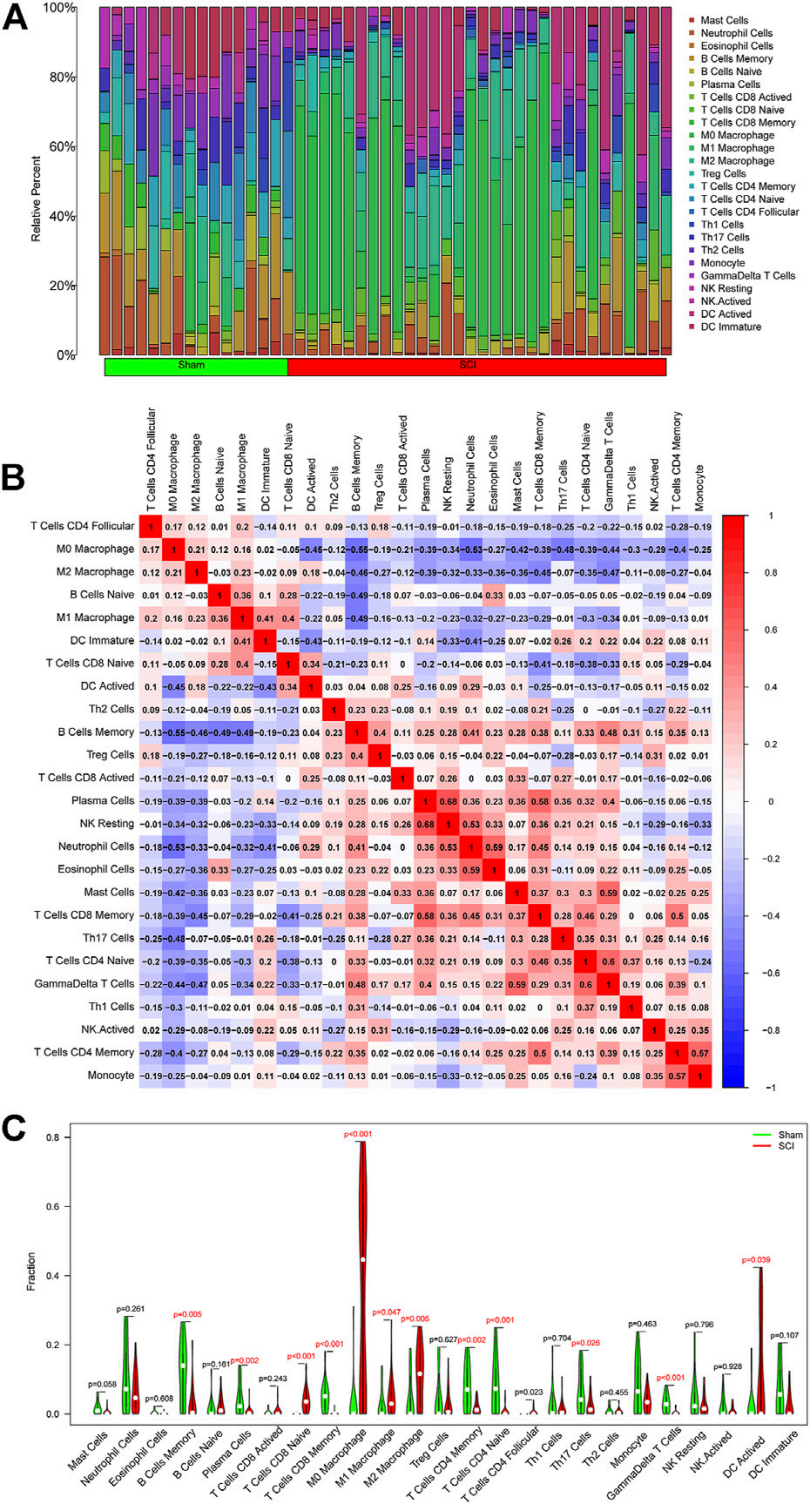
Assessment and visualization of immune cell infiltration. **(A)**The relative percentage of 25 types of immune cells. **(B)** Heatmap exhibited the correlation of 25 types of immune cells. Both horizontal and vertical axes demonstrate immune cell subtypes. **(C)** The violin diagram displayed the ratio of 25 types of immune cells. The red represents the SCI group, and the green represents the sham group.

To further investigate the association between SCI and immune cells during the development of SCI, we predicted immune cell infiltration using the CIBERSORT method. Figure 6A is a bar plot depicting the percentages of the 25 different kinds of immune cells. As revealed by the correlation heatmap for the 25 different types of immune cells, memory B cells and M0 macrophages, we found that neutrophil cells and M0 macrophages exhibited a substantial negative association. We also found substantial positive associations between monocytes and memory CD4 T cells, γδ T cells and naive CD4 T cells, γδ T cells and mast cells, eosinophils and neutrophils, natural killer resting cells and plasma cells, and memory CD8 T cells and plasma cells (Figure 6B). Furthermore, the SCI group had significantly higher proportions of naive CD8 T Cells, CD4 Follicular T cells, M0 macrophages, M1 macrophages, M2 macrophages, DC-activated cells than the sham group, and markedly lower proportions of memory B cells, plasma cells, memory CD8 T cells, memory CD4 T cells, naive CD4 T cells, Th17 Cells, and γδ T cells (Figure 6C).

**FIGURE 7:**
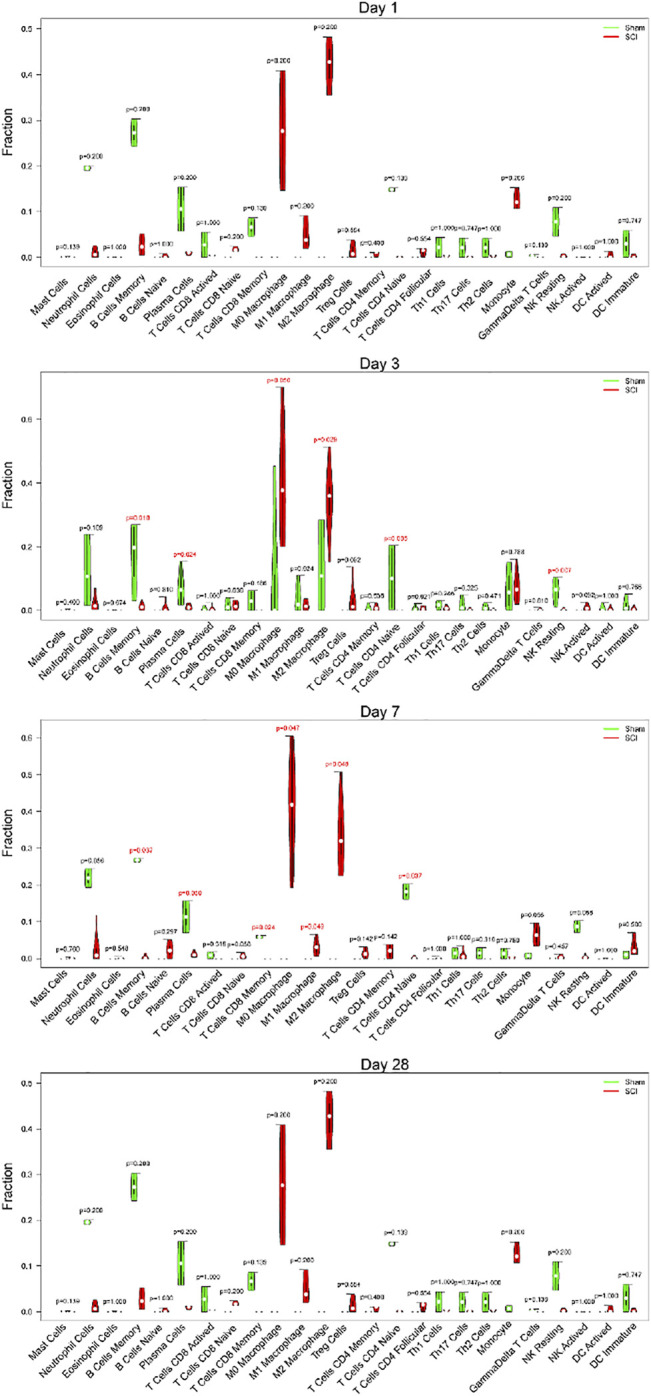
Different time points of immune cell infiltration. The violin diagram exhibited the ratio of immune cells at different time points (1, 3, 7, 28 days of post-injury). The red represents the SCI group, and the green represents the sham group.

Immune cell infiltration gradually played an important role after SCI. Thus, we selected different time points (1, 3, 7, 28 days of post-injury) to show the relationship between SCI and immune cells. We found no obvious difference between the SCI group and the sham group on the first day. However, the SCI group had significantly higher proportions of M0 macrophages, M2 macrophages than the sham group, and markedly lower proportions of memory B cells, plasma cells, naive CD4 T cells, and NK resting cells at day 3. At 7 days, the SCI group displayed higher ratios of M0 macrophages, M1 macrophages, M2 macrophages than the sham group, and noticeably lower ratios of memory B cells, plasma cells, memory CD8 T cells, and naive CD4 T cells at day 7. On day 28, there was no apparent difference between the SCI group and the sham group (Figure 7).

**FIGURE 8:**
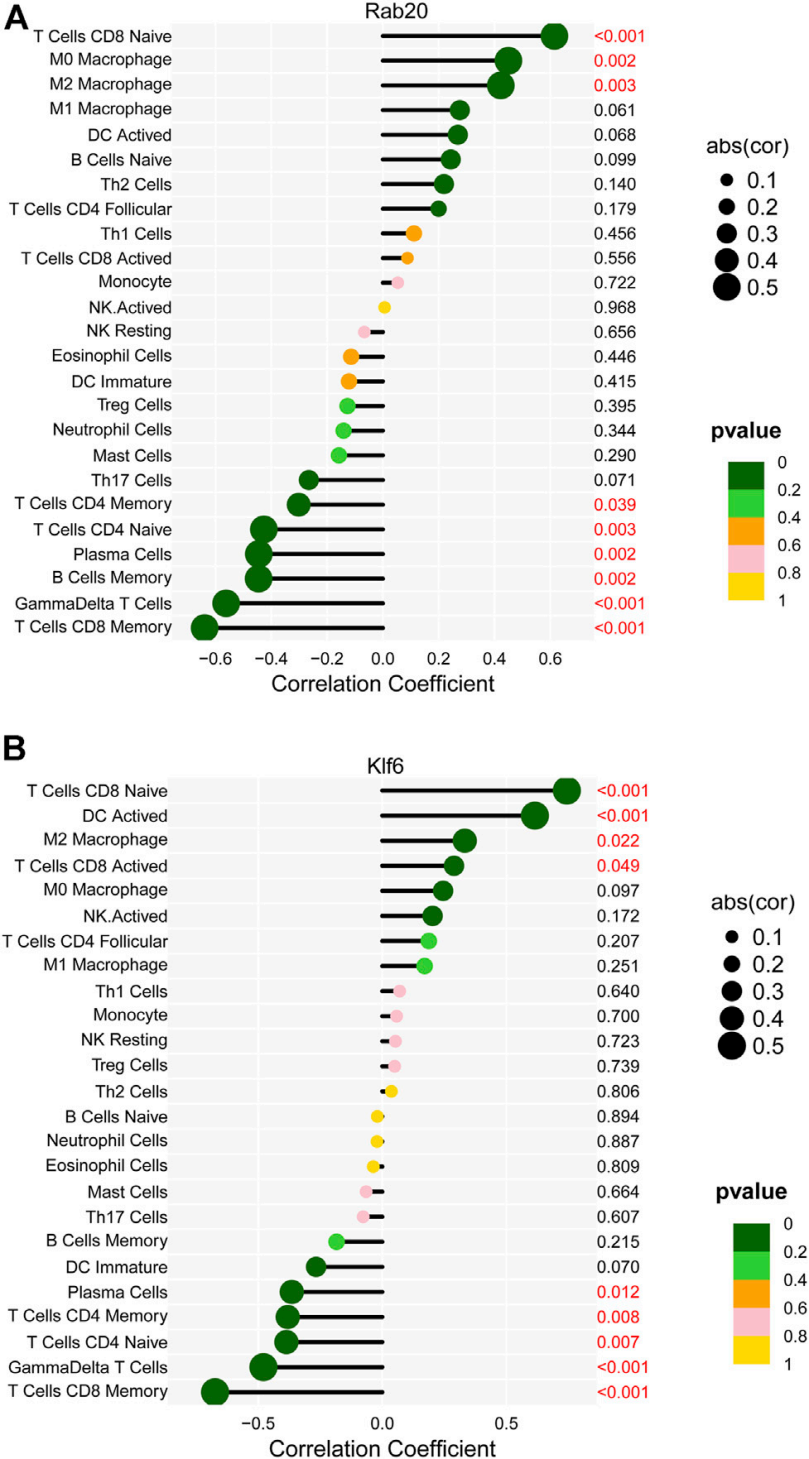
The Correlation between key markers and infiltrating immune cells. **(A)** Correlation between Rab20 and infiltrating immune cells. **(B)** Correlation between Klf6 and infiltrating immune cells.

Based on the correlation analysis, Rab20 was positively correlated with M0 macrophages, M2 macrophages, and naive CD8 T cells and negatively correlated with memory CD4 T cells, naive CD4 T cells, plasma cells, memory B cells, γδ T Cells, and memory CD8 T cells (Figure 8A). In addition, Klf6 was positively correlated with naive CD8 T cells, DC-activated cells, M2 macrophages, and activated CD8 T cells and negatively correlated with plasma cells, memory CD4 T cells, naive CD4 T cells, γδ T cells, and memory CD8 T Cells (Figure 8B).

## Discussion

SCI frequently results in permanent functional deficits below the affected spinal cord region. The pathology of SCI is generally divided into two processes, named the primary injury and secondary injury. The secondary injury plays a crucial role in SCI onset and progression, leading to acute and chronic inflammation, tissue architecture damage, and motor and sensory dysfunction (Kong and Gao, 2017). Additionally, current research indicates that immune cell infiltration noticeably affects SCI development and progression (Al Mamun et al., 2021). Therefore, this study aimed to discover relevant SCI biomarkers and to investigate the role of immune cell infiltration in SCI.

Based on the GEO database, researchers can easily access Spinal cord injury (SCI) related datasets. Two SCI datasets (GSE5296, GSE47681) were included in this study (Zhao et al., 2018; Liu et al., 2019; Wei et al., 2019). We identified 561 DEGs in total. Among them, 536 were up-regulated and 25 were downregulated. Next, we performed functional enrichment analysis on these DEGs and found potential associations with immune responses and inflammatory signals (e.g., regulation of inflammatory response, leukocyte migration, positive regulation of cytokine production, and cytokine-mediated signaling pathway). Furthermore, The top 10 pathways of these DEGs according to *p* value were screened. Cytokines are crucial for immune response, pro-inflammatory cytokines influence the progression of disease (Bass et al., 2008). Cytokine–cytokine receptor interaction can be activated by neuroinflammation after SCI (Baek et al., 2017). Phagocytosis has important functions in immunity. Innate immune cells recognize and degrade microbes and debris by phagosomes. Macrophages process the debris of the spinal cord and promote the neurological function recovery after SCI (Zhou et al., 2020). TNF signaling pathway involves in the modulation of immune response and triggering the activation of T cells to induce cell death. The suppression of the TNF-α signaling pathway promotes function restoration after SCI (Wang N. et al., 2018). IL-17 is a pro-inflammatory cytokine and generated by T helper 17 cells (Torchinsky and Blander, 2010). IL-17 exacerbates the neuroinflammation of the spinal cord after SCI in the rat (Zong et al., 2014). Atherosclerosis is a chronic inflammatory disease. Monocytes and macrophages contribute to the initiation and development of atherosclerosis (Moroni et al., 2019). Osteoclasts originated from hematopoietic monocyte-macrophage lineage. Osteoclast differentiation is mainly regulated by receptor activators of NF-κB and immune receptors (Park-Min et al., 2009). The pathology of tuberculosis is closely related to immune cells. Innate immune cells determined the inflammatory environment against Mycobacterium tuberculosis infection and induced adaptive immune responses (de Martino et al., 2019). Rheumatoid arthritis (RA) is a systemic inflammatory disorder. Immune cells (like T-cells, B cells, and macrophages) played a crucial role in the pathogenesis of RA (Yap et al., 2018). Viruses have produced many mechanisms to escape detection and destruction from the immune system by copying and repurposing host cytokine and cytokine receptor genes. Viral protein interaction with cytokine and cytokine receptor activates downstream cytokine signaling and affects different immune processes (Bruggeman, 2007). Immunity and leishmaniasis are also closely related. Leishmania first infected macrophages in the host. Then neutrophils secreted chemokine (C-C motif) ligand 3 (CCL3) to recruit dendritic cells. The interleukin (IL) 12 was produced by dendritic cell, which induced the differentiation of T helper type (Th) 1 cell to produce more IFN-γ to control the infection of Leishmania (de Freitas and von Stebut, 2021).

To explore the potential biomarkers during the development of SCI, we integrated and analyzed two mouse SCI datasets. We only selected SCI-related data. Thus, 47 samples were included. Based on the differentially expressed genes (DEGs), three machinery learning methods were applied for screening important genes. The random forest (RF) is a non-parametric approach for achieving classification under supervision. The term “random forest” refers to decision trees constructed from a subdivided data set. This method does not generate overfitting phenomena readily and exhibits strong anti-noise properties (Yang et al., 2020). Thus, the RF method has been employed widely in recent years for prediction. ASSO logistic regression is a comprehensive machine-learning method for selecting variables by identifying those with the lowest chance of classification error. SVM-RFE is a machine learning approach for ranking and selecting the most significant features for classification. Every method obtained some essential genes. This study integrated these three distinct methods. We picked Rab20 and Klf6 because they were overlapped genes. In a previous study, researchers found a possible link with the immune and inflammatory functions, neuronal function, and synaptic transmission based on the functional enrichment analysis (GO and KEGG) of differentially expressed genes (DEGs) from GSE5296. Then they defined and collected these Neuronal function and synaptic transmission-associated genes and inflammation-associated genes from the literature review and investigated their expression in trauma site (R), adjacent rostral (M), and caudal (C) regions at different time points after SCI (Chen et al., 2015; Zhao et al., 2018). Another study aimed to explore the critical genes with genes expression of SCI from trkB.T1 knockout mice. This study identified the top four modules genes from GSE47681 using Weighted correlation network analysis (WGCNA). These module genes were used to construct the Protein-protein interaction (PPI) network. Finally, protein tyrosine phosphatase, receptor type C (PTPRC), coagulation factor II, thrombin (F2), plasminogen (PLG) were the most significant nodes in the PPI network (Wei et al., 2019). Compared to these two studies, we confirmed the differential expression of Rab20 and Klf6 with validation experiments, whereas other did not. Secondly, we used different screening methods to obtain SCI-related biomarkers. Importantly, our combined approach is more innovative as it points straightforward to relevant SCI markers Rab20 and Klf6, which are still not that much investigated.

Rab20 is a member of the Rab GTPase family, associating with macropinosomes at stages that overlap with those of Rab5, Rab21, Rab7, and Lamp1. Rab20 up-regulation may contribute to plaque destabilization *via* increased autophagy and cell death (Cederstrom et al., 2020). High Rab20 levels promote B cell activation and facilitate rheumatoid arthritis development (Tseng et al., 2019). Rab20’s expression was increased during B cell transformation by a polymorphism associated with Crohn’s disease and vaccination (Mehta et al., 2017). Additionally, Rab20 is an interferon-regulated Rab GTPase that promotes the homotypic fusion of early endosomes and directs endosomal cargo to lysosomes for degradation (Pei et al., 2015).

The Klf family of zinc finger transcription factors participates in various processes, including development, cellular differentiation, and stem cell biology. Klf6 promotes corticospinal tract sprouting and regeneration after SCI (Kramer et al., 2021). Alternatively, Klf6 is required for chronic pain maintenance, emphasizing its potential as a therapeutic target in chronic pain management (Mamet et al., 2017). When expressed ectopically in the adult injured central nervous system, Klf6 can promote axon growth (Wang Z. et al., 2018). Therefore, the identification of Rab20 and Klf6 together may imply that Rab20-mediated phagosomes cause cell death and KLf6 promotes nerve regeneration during SCI.

To more precisely assess the impact of immune cell infiltration in SCI, we analyzed immune cell infiltration through mice tissue expression profiles using CIBERSORT (Chen et al., 2017). The immune cell infiltration of M1, M0, and M2 macrophages, naive CD8 T cells, follicular CD4 T Cells, and DC-activated cells increased, indicating possible associations with SCI development and progression. Additionally, immune cell infiltration may have the property of dynamic changes at different time points after SCI. Microglia are well-known as the central nervous system’s resident immune cells. After traumatic SCI, microglia/macrophages and neutrophils are recruited to the damaged location (Xu L. et al., 2021). Macrophages, microglia, and other antigen-presenting cells (APCs) activate T lymphocytes. SCI inhibits B cell activation and antibody production (Ankeny and Popovich, 2010). Autoantibodies aggravate post-SCI complications such as cardiovascular, renal, and reproductive failure (Alizadeh et al., 2019). Although this has been mentioned previously, more investigation into the molecular mechanisms and effects of immune cell infiltration in SCI seems required.

Regarding the associations between immune cells and key genes, Rab20 was positively correlated with M0 and M2 macrophages and naive CD8 T cells during activation and negatively correlated with memory CD4 T cells, naive CD4 T cells, plasma cells and memory B cells, γδ T cells, and memory CD8 T cells. Further, Klf6 was positively correlated with naive CD8 T cells, DC-activated cells, M2 macrophages, and activated CD8 T cells, but negatively correlated with plasma cells, memory CD4 T cells, naive CD4 T cells, γδ T cells, and memory CD8 T cells. According to one study, the expression of a dominant-negative Rab20 mutant may impair macrophage phagosome maturation (Pei et al., 2014). KLF6 promotes HIF1 expression in macrophages, regulating inflammatory and hypoxic responses (Kim et al., 2019). Because there is no additional information about the sophisticated interaction mechanisms between key genes and immune cells, they should be thoroughly investigated based on the assumption mentioned above.

We used novel and scientific approaches (e.g., LASSO logistic regression, random forest, and SVM-RFE algorithm) to identify characteristic SCI makers. Additionally, we used CIBERSORT to investigate immune cell infiltration. Nonetheless, this study has some limitations. First, it is the result of the second round of data mining and analysis. Additionally, we did not obtain clinical specimens for this study and had to rely on mouse tissue to confirm our predictions. Finally, the results’ reliability should be thoroughly validated using large samples.

## Data Availability

The raw data supporting the conclusions of this article will be made available by the authors, without undue reservation.
